# Sexual and gender minority undergraduates’ relationships and strategies for managing fit in STEM

**DOI:** 10.1371/journal.pone.0263561

**Published:** 2022-03-17

**Authors:** Rebecca Campbell-Montalvo, Mya Malaykhan, Chrystal A. S. Smith, Michelle Hughes Miller, Ellen Puccia, Maralee Mayberry, John Skvoretz, Hesborn Wao

**Affiliations:** 1 Department of Curriculum and Instruction, Neag School of Education, University of Connecticut, Storrs, Connecticut, United States of America; 2 Department of Anthropology, University of Connecticut, Storrs, Connecticut, United States of America; 3 Department of Women and Gender Studies, University of South Florida, Tampa, Florida, United States of America; 4 Beta Research Associates, Inc., Palmetto, Florida, United States of America; 5 Department of Sociology, University of South Florida, Tampa, Florida, United States of America; Hackensack Meridian Health, UNITED STATES

## Abstract

Undergraduates with sexual and/or gender minority (SGM) identities, including lesbian, gay, bisexual, transgender, queer/questioning, pansexual, intersexual, asexual, or additional positionalities, often face an unwelcoming STEM microclimate. The STEM microclimate includes the places students experience, such as classrooms or labs, and the people, such as peers or professors, with whom they discuss their STEM program. While previous work offers a framework of microaggressions faced by SGM people, and the behavioral, cognitive, and emotional strategies they use to react to them, little is known about the strategies SGM students use to persist in the STEM microclimate. We analyze interviews with 29 SGM STEM undergraduates to uncover how they fit in STEM, their experiences that affect fit, how social capital in the form of influential others affects fit, and the strategies used to deal with microaggressions and cultivate a supportive network. Using thematic analysis, we find that students vary in their feelings of fit, with students with gender minority identities experiencing more frequent and more severe microaggressions than students with sexual minority identities (which are often less visible). We likewise find that students with racial minority identities report compounding issues related to identity. SGM students with social capital, or a network of people to whom they can turn in order to access advice and resources, believe they fit in better than those without such capital. To support their feelings of fit, students use defenses against discrimination, including micro-defenses, wherein they change how they present their self to avoid microaggressions and/or surround themselves with accepting people. This research highlights the role of microaggressions and social capital in affecting fit as well as the micro-defenses students use to defend against discrimination. Our introduction of the concept of micro-defenses provides a way to theorize about micro-interactional dynamics and the site at which students defend against microaggressions so they feel more welcome in STEM. Implications provide insight into how SGM students can be supported in STEM as well as the institutional changes STEM departments and campuses can make in order to better support and include SGM students.

## Introduction

Sexual and gender minority (SGM) students identify as Lesbian, Gay, Bisexual, Transgender, Queer/Questioning (LGBTQ) or additional sexual or gender minority positionalities [1]. The number of SGM students in STEM is unknown, though the number of undergraduates who identify as SGM is increasing [2, 3]. In general, SGM students are at “highest risk for experiencing” behavior from others “that interferes with their ability to live and learn on campus” [4, p. 11]. STEM undergraduate programs and environments are typically founded upon hetero- and cis-normative standards and can be unwelcoming of other gender identities and sexualities [5–8]. Specifically, SGM students often deal with discrimination and exclusion due to others’ perceptions, biases, and stereotyping of their minoritized sexual and gender identities [5, 9, 10]. This treatment is frequently in the form of microaggressions—covert, indirect, restrained, and ambiguous demonstrations of discrimination and prejudice against minoritized groups [6, 11, 12], such as cis-normative language use by faculty [13], including their resistance to trans or non-binary students’ pronouns [14, 15]. This environment affects the lower STEM major declaration rates, degree persistence trends, and workforce representation of SGM people in comparison to their non-SGM counterparts [8, 16–23].

To understand how SGM students persist in STEM, we examine the effects of these microaggressions and social capital on their feelings of belonging or inclusion (i.e., fit) in STEM. In our examination of social capital, we specifically focus on SGM students’ relationships with peers and others in their social networks known as ‘alters’ in and outside of STEM. Our work is situated within the curricular space known as the STEM microclimate—the entire space within which discussions and activities related to students’ STEM majors take place [24–26]. Previous work has demonstrated an unwelcoming climate with biases against women and underrepresented racial minority students in STEM [6, 27–32] and generally [33]. However, accessing and activating social capital can help students cope with feelings of isolation and improve fit [29, 32, 34]. To our knowledge, social capital theory, precisely social networks, have not been previously explored qualitatively in an SGM STEM population [see 35].

The primary contribution of this study is its focus on how SGM STEM undergraduates manage the generally unwelcoming STEM microclimate through a variety of purposive self-protective measures and behaviors, including those that are behavioral, cognitive, and emotional [13], such as by making micro-interactional changes in presentation of self (e.g., clothing, language, signs) and cultivating a social network of supportive people. By uncovering the work these students must do to persist, we highlight the existing STEM microclimate, underscoring how it can be changed to encourage persistence. In this study we draw on interviews with 29 SGM undergraduate STEM majors to answer the following research questions:

1. What are SGM STEM undergraduates’ perceptions of how they fit into their degree programs?
2. What experiences and interactions, including microaggressions, influence their perceptions of fit?
3. In what ways does having or not having supportive alters influence SGM STEM undergraduates’ perceptions of fit?
4. What strategies have SGM students employed to deal with stereotypes or microaggressive language or acts from others and enhance their fit in STEM?

In the following sections we review literature on academic climate, social capital, and resilience to situate the present work, explain the methods for data collection and analysis of the interviews, and present our findings related to the research questions from this study. We end with a conclusion identifying potential applications of this work and future research opportunities.

## Literature review

### Academic climate’s effect on fit for SGM students

Academic climate in the STEM literature refers to both psychological climate (student perceptions of others’ attitudes, behaviors, and practices) and experiential climate (student experiences of discrimination, harassment, and abuse) [36]. Microaggressions are often unrecognized by non-minoritized groups [11], and these experiences can lead to SGM students experiencing poor climate, which can in turn impact how they feel they belong or fit in their program. We use ‘fit’ to refer to how students feel they belong or feel welcomed into their STEM programs [see 32, 34, 35]. We view fit as a systematic characteristic, reflecting their STEM program culture, not students’ own supposed weaknesses or strengths. We interpret fit as nestled within students’ cultural models of education—or how they conceptualize and understand education and the people within the schooling process [37, 38].

Within the STEM microclimate, there can be differing climate zones, or various places in which academic climate and thus fit can differ, such as in departments or labs [29], and students’ feelings of fit may not be uniform across all places in the microclimate. Nadal and colleagues [33] offer a taxonomy of microaggressions against SGM people, including the use of and endorsement of cis-normative and heterosexist language and behaviors and the denial that social and individual prejudice and discrimination against SGM people occurs. In STEM, classroom interactions can be particularly fraught sites of cis-normative language [39]; transgender students have reported faculty refusal to use appropriate pronouns in direct address, which can lead to student disengagement from courses [14].

These findings demonstrating the negative environment in STEM for SGM students are bolstered by several other studies [40–42], such as Bilimoria and Stewart who document that heterosexuality is often assumed by others in STEM [43] or Woodford and colleagues who show how hearing the phrase “That’s so gay” impacted students’ social and physical well-being [44, 45]. STEM departments are suggested to be chilly in comparison to others (such as social sciences) that are warm to SGM students [21, 26, 42, 46]. Differences among STEM fields, such as the life sciences and physical sciences, in biases against SGM students also likely exist given the demonstrated differences between the fields in biases against women [27]. For instance, the hegemonic masculinity culture of engineering has long been documented to affect women [32, 47–49], with work highlighting the importance of hostile experiences related to masculinity for students who were gay men and stereotyped as feminine to a degree that being gay was seen as a barrier to success in engineering [50]. Together, these studies show a general pattern in STEM programs where SGM students tend to experience microaggressions related to sexual and gender identity, causing negative STEM microclimates and likely poor fit.

### Social capital: The effect of SGM alters on fit and persistence

The lack of SGM faculty, alters, mentors, and students within STEM fields can additionally contribute to feeling a lack being welcomed or fitting in for SGM students [25]. Thus, we consider how SGM students’ alters affect how they fit into their STEM program. In our work, we rely on Lin’s characterization of social capital, in which he suggests that access to resources comes from ties that link an individual to others with resources [51]. Further, Coleman suggests social capital is a ‘collective’ resource or group asset that results from societal structure [52]. Specifically, social capital is “the norms and networks that enable people to work collectively to mobilize resources and achieve common goals” [53, p. 111].

Most recently, in conceptualizing types of alters, Skvoretz and colleagues describe network-based social capital as emphasizing an individual’s network of social ties, while participatory social capital is accessed by an individual’s participation in organizations that have as part of their mission to support students [32, 54, 55]. Social networks built through both of these types of relationships offer multiple forms of support that relate to academic degrees and careers, including subject knowledge, psychological and emotional support, and role modeling [55–57]. Research shows that alters can provide valuable advice to women and underrepresented minority students in engineering to help them cope with microaggressions, fit, and a range of social and academic concerns [32, 55, 57]. Recent work has shown that professional STEM societies which focus on SGM students (e.g., *o*STEM) provide crucial emotional and support resources to SGM students that help them navigate reactions to their identities and fit in STEM [35]. Importantly, social capital research emphasizes the ways in which socio-demographics (e.g., gender, sexuality, ethno-racial grouping, age, education) affect network position.

Because they are perceived as traditional outsiders, SGM individuals, especially if they are “out”, might have less access to benefits from network-based social capital [29, 58, 59], which could contribute to poorer feelings of fit. In particular, not all transgender students receive the same level or quality of faculty mentoring as that received by their cisgender peers [60]. SGM students sometimes find it difficult to connect with faculty mentors who also can be role models [61, 62]. Research offers differing findings relating to professors and SGM students, with some noting that many faculty are “clueless” when it comes to assisting SGM students [43], while others are more clearly supportive [42]. Student peer relationships also affect their persistence and retention [63]. The amount of, and degree to which, students have positive interpersonal interactions with peers can predict their success as well as their intellectual and personal development [64]. Negative peer relationships, as the most likely source of harassment and exclusion experienced by SGM students, also impact their persistence [14, 65]. Previous research has shown that STEM environments with more women are more welcoming to SGM identities [66], but this may have little overall impact, as STEM departments tend to lack women students and faculty. In terms of participatory social capital, outreach to SGM-focused organizations (e.g., *o*STEM) may be very important for SGM undergraduates, who frequently report the need to establish relationships with faculty, staff, and peers outside their discipline [26, 55, 65]. Such organizations may create a physical or epistemological safe space outside students’ academic departments [26, 67]. These organizations are shown to potentially more often offer support to help students persist by focusing on emotional support (e.g., helping students make sense of others’ reactions to their identities), rather than providing direct academic support and resources (e.g., advice on how to study) [35].

### Resilience in STEM: Using micro-defenses to fit

Nadal and colleagues [33] classified the ways that SGM people react to microaggressions in three ways. These ways include behavioral reactions or how SGM people react to microaggressions in terms of actions (such as through ignoring the microaggressions or confronting others about them), cognitive reactions or how people think about or make sense of microaggressions (such as through accepting them or becoming resilient in dealing with them), and emotional reactions or the emotions people experience as a result of the microaggressions (such as feeling sad or angry). Research on cultural norms in engineering programs has highlighted the behavioral, cognitive, and emotional efforts required by SGM students to express identity or ‘navigate’ [68] engineering programs’ heteronormative climate (e.g., passing, living compartmentalized lives, and isolating oneself from engineering peers) which entail a tremendous amount of emotional work and can “limit these students’ opportunities to succeed, relative to their heterosexual peers” [7, p. 1]. Our study focuses on SGM students in STEM and primarily their behavioral reactions, though emotional and cognitive reactions also emerged in our work, in which students employed various strategies to manage identity and cultivate a network of supportive alters.

Recent years have seen more work on SGM students in STEM, with the most recent studies paying attention to students’ cognitive reactions or resilience as well as their social capital rather than simply their academic-climate related obstacles. For instance, Kersey and Voigt found that SGM students’ sense of resilience was usually combined with a deep desire to pursue STEM, that students persisted in STEM as a form of resistance, and that professional SGM societies were a powerful support for student resilience [46]. Miller and colleagues’ work on SGM STEM persistence focused on the behavioral and cognitive strategies students used to navigate the hypermasculine “Dude” or “Bro” culture in STEM, including participating in it, resisting it, traversing liminal parts of it, blending into it, or rationalizing it [69]. Similarly, Steele found that gay men used behavioral strategies like closetedness and gender performativity to deal with the unwelcoming STEM environment [41]. This finding replicates Cech and Waidzunas’ earlier work suggesting that passing and covering (e.g., closetedness) were reported strategies by SGM in STEM [7]. The authors additionally found that SGM students mobilized skill development to make themselves indispensable to others, for example, by mastering mapping programs so that they would be valuable group members on teamwork assignments. Finally, Mattheis, Cruz-Ramírez De Arellano, and Yoder used a cognitive queer STEM identity framework to understand how SGM STEM employees “come to understand and name themselves as queer in terms of gender and/or sexuality,” form specific STEM identities, and navigate professional and personal influences on how they express identity in work and school [68, p. 1850]. The authors primarily focused on internal identity work wherein participants compartmentalized aspects of their identity and put less focus on their strategies for mitigating fit, though they do briefly mention behavioral strategies such as being more visible, creating changes in work environments, and seeking out SGM networks as coping mechanisms.

To better understand how students navigate STEM academic climates, our study builds on these intersecting bodies of literature that highlight SGM behavioral strategies of persistence by introducing the concept of micro-defenses. The concept of micro-defenses extends work on emotional strategies of resilience in STEM by offering a concept that is focused on the on-the-ground, external micro-interactions within the STEM microclimate that also brings together work on behavioral strategies and social networks. The term micro-defense is similar to Sue and colleagues’ [70] concept of microintervention, with the former focusing on the strategies minoritized students use to navigate microaggressions in the STEM climate as a form of social capital and the latter offering a broader conceptualization taking into consideration not only those who are affected (i.e., targets) but also allies and bystanders. Additionally, Sue and colleagues’ framework offers an expansive articulation of the multiple reasons and purposes for which a microintervention might be used (e.g., to educate the offender) that go beyond the focus of STEM degree persistence in our work here. We also extend Nadal’s work [13] by offering a specific mechanism through which behavioral, cognitive, and emotional strategies are all employed by SGM students as they persist in STEM.

To be clear, within the STEM microclimate, micro-defenses are specific, individual interactional processes through which students deploy a range of behavioral, emotional, and cognitive defenses, such as stereotype management or building a social network of supportive alters [30]. Micro-defenses oppose microaggressions, both as reactions and preemptively. When a microaggressive act or comment is made, a comment or act made in resistive response to it is a micro-defense. Micro-defenses can also occur without being immediately precipitated by a microaggression, for instance, when done in order to prevent microaggressions (e.g., a student affixing a ‘PRIDE’ sticker to their laptop and then using their laptop in STEM spaces to make a statement about acceptance of or identities related to gay rights or a student choosing not to affix the sticker to avoid potentially negative reactions of others in class or lab). Importantly, micro-defenses can also include the cultivation of social networks to include supportive others. This cultivation supports the pre-emptive avoidance of unsafe people who would not be supportive of SGM students’ identities. These networks would fulfil the needs of SGM students who encounter a negative STEM microclimate, while supporting their access to resources to achieve their STEM goals.

## Method

The University of Connecticut Institutional Review Board approved this study (#H17-188) on 11/14/2017.

### Recruitment, participants, and measures

The research team conducted interviews with 29 SGM STEM undergraduates recruited through two universities and six participating national STEM professional organizations. To facilitate recruitment, the organizations distributed IRB-approved recruitment statements to their members, while project personnel at the two universities distributed IRB-approved recruitment emails to local SGM and STEM group leaders. Organization members and university group leaders then distributed the recruitment statements through their own networks.

Individuals interested in participating in the study contacted the PI to schedule interviews. They completed an information form (S1 Protocol) that asked about their gender identities, sexual orientations, races/ethnicities, academic background, and their academic and career goals. They uploaded the form using a secure link provided by the researchers. The research team then contacted participants to schedule videoconference interviews via Skype or Zoom. Two research team members co-interviewed each participant. There were two two-member teams led by a total of four researchers. Participants received a $25 incentive for participating in the study. As shown in Table 1, the 29 participants in this study self-identified a range of gender identities, sexual identities, and races/ethnicities. Participants were able to select all gender, sexual, and racial/ethnic identities applicable to them.

**Table 1:** Participant characteristics.

Gender Identities	#	Sexual Identities	#	Race/ Ethnicity	#
Agender	1	Asexual	3	Asian	7
Man	6	Bisexual	10	Black/African American	2
Non-Binary	1	Gay	9	Other	1
Non-Binary/Queer	3	Lesbian	7	White	19
Transgender	4	Queer	7		
Woman	17				

After refining the interview protocol (S2 Protocol) guided by the analysis of six transcribed pilot interviews and feedback from advisory committee members, the interviewers were trained on and used the same interview guide and protocol. The interview protocol focused on 1) student’s perceptions of their fit in their STEM microclimate; 2) how students managed their gender and/or sexual identities within their STEM microclimate; 3) how students navigated the STEM microclimate, including their experiences of microaggressions; and 4) students’ social capital (defined as students’ social networks, or people influential to them in their pursuit of their degrees). The interview guide focused on the STEM microclimate, but we also include responses here wherein students were asked about the STEM microclimate but occasionally discussed other factors (e.g., roommates) outside of the immediate STEM microclimate that nevertheless affected how they persisted in STEM. In addition, the interview guide predominantly contained items to ascertain how students’ SGM identities affected their fit in STEM, whether those identities mattered during interactions in the STEM microclimate, and how they navigated such spaces in light of their identities. Sample items from the interview protocol include:

- “As an individual who is [insert their identities reported on demographic form], how do you feel like you fit in your STEM program?”
- “Do you think any of your identities have mattered to your peers/instructors/mentors in your STEM program? In what ways?”
- “How have others’ perceptions of or reactions to your identities and experiences affected your choices while pursuing your STEM goals?”
- “Tell me about the kind of information and resources you have accessed through these relationships to assist you in pursuing your STEM goals.”

Interviews lasted approximately one hour and were audio recorded and transcribed verbatim. Each interview began with the informed consent process followed by the interviewers reviewing the demographic form that was sent out ahead of time to confirm the participants’ identities.

### Analysis

A total of five members of the research team engaged in the analysis that informs this article. First, four core members of the team read the 29 transcripts several times and engaged in several rounds of conversation about how the interviews informed the project goals. These conversations then spurred a focus on the variables presented in this study. Interviews were analyzed using thematic analysis in which a codebook was developed based on these conversations by two of the four core members of the research team. The codes related to the key concepts of this project, including the concepts of fit and social capital [71]. The codes analyzed for this article included: how participants felt they fit in their STEM program, how they managed their fit in STEM, the role of language in STEM fit, and the role of the institution in STEM fit. These codes did not necessarily coincide with specific interview questions, but rather were used based on the major concepts in the literature and the research team’s review of the transcripts and familiarity with their content. The same two members of the research team, along with a fifth member, held several meetings in which the codes were discussed and created, including the definitions of each code. As part of these discussions, it was clear that lines of data could possibly fall into more than one code, in which case it was determined that all appropriate codes should be used. The fifth member of the research team imported the transcripts into NVivo, a qualitative data analysis computer software used for the analysis of qualitative data, and then read each transcript and applied the codes within the program.

Coding was done in a wide purview, meaning that codes were applied to include the interview question and the entire passage of interest so that context could be retained for analysis. Two members of the research team then exported all the passages associated with each code and reviewed the excerpts linked to each of the codes one at a time in order to identify themes related to the key concepts of this work within the students’ responses. Specifically, aside from organizing this initial round of coding, we did not use NVivo for additional coding. Instead we used analytic memoing to identify trends or patterns and the relationships among these patterns. We also identified and analyzed unique experiences reported by our respondents in relation to our research questions from the exported passages to ascertain their relationship to respondents’ status characteristics or other experiences.

Our analytic memos were then collectively crafted into the themes presented here [72]. The five-person research team discussed and circulated drafts of the themes and refined them based on the literature and familiarity with the data. No themes were excluded or discarded, but some were revised to highlight particular foci (i.e., we highlighted students’ reactions to microaggressions as micro-defenses, though portions of that theme could have also been located within the earlier section on climate and fit). After finalizing these themes, quotes taken from the coded excerpts were compiled for demonstration, and then were organized in relation to the research questions. In the end, the team reached consensus about how well the themes represented the interview data overall, thus demonstrating the reliability of our data. Our findings are credible given that the interview guide was based on questions and findings from previous research, transferable as we engaged in appropriate sampling procedures and have a diverse sample of participants (including those from a range of universities and programs), confirmable given that the findings come from our analysis process articulated above and participants’ own words, and dependable as we expect that researchers studying SGM students in STEM will likely find similar student experiences and strategies in their data [73].

## Results

Students’ perceptions of fit in their STEM departments varied, but majority statuses often mediated the STEM microclimate for students who can “pass” as straight or masculine. Indeed, our results show the multiple factors that affected a STEM student’s perceptions of fit, such as sexual and gender stereotypes, wearing clothing that is considered by traditional norms as lacking gender-conformity, and using preferred pronouns. Students noticed that they felt more comfortable with peers and faculty who were women or people of color, and they appreciated having SGM students and faculty to share their experiences. Therefore, SGM students actively engaged in micro-defenses in which they hid or presented their selves differently in various microclimates, only shared information with others they deemed trustworthy, actively pursued relationships with women professors and faculty of color to expand their social networks with supportive others, and cultivated a comfortable microclimate in STEM that would help them achieve their STEM goals. Note, in the excerpts presented in this results section, we use the pronouns selected by each respondent and pseudonyms to ensure participant confidentiality.

Importantly, this research represents an initial examination of the relationship between these variables using cross-sectional interviews. As we show, the data suggest that strong relationships with alters increased students’ feelings of fit and absent alters decreased their fit. Design limitations urge caution in interpreting causality—it is possible that students who fit well were more able to cultivate social capital, a possibility given that poor fit has been shown to inhibit students’ ability to gain social capital [74].

### Perceptions of fit: Majority statuses offer intersecting protection

Twelve of 29 students (41%) reported fitting in well in their STEM department. All of these students were sexual minorities, and none were gender minorities. These students described their fit similarly; they reported that they felt comfortable, safe, and well liked and never felt excluded in their STEM microclimate. For example, when asked if he had ever felt excluded in his STEM department, Jeff, a white gay man, said, “Not really, to be honest…[the communities] I am a part of, whether it be from school or work, have all been pretty including.” Similarly, Jess, a white bisexual woman, believed her identities did not really impact her relationships with professors. She commented, “Because of the fact that [my bisexuality] was not as visible to mentors and faculty members, I don’t think it’s made much of an impact.” In another example, Kevin, a white gay man, stated that others’ reactions to his majority statuses and the hidden visibility of this sexuality promoted his feeling of fit in STEM. He noted:

I think that’s very much because I can be straight passing. It would be harder if I wasn’t as straight passing or if I wasn’t male or white. But for me personally, I haven’t ever felt excluded from those communities.

Seven students (24%) said they fit but noted that there were varying circumstances or climate zones (i.e., locations) affecting their fit at particular times. Michael, a white gay transgender student, felt safe in their department, for instance, but not on the campus as a whole. Some students reported fitting in with regards to some of their identities, but not others. For instance, Lina, a bisexual Asian woman, noted that she fit on campus but felt “very out of place” in STEM and that her identity often stood out because of her intersecting gender, racial, and sexual identities. Lina commented, “I’m the only girl and only Asian and only gay person [in my STEM department].” Similarly, three other women said they felt they fit, but at times their identity as a woman made them feel uncomfortable or unsafe. For example, when asked how biases against women have affected her comfort and safety in her department, Kylie, a white lesbian woman shared, “It makes me feel a lot less safe in the field knowing that my emotional health could potentially be compromised by going into a STEM field [because of my gender].” Here, Kylie connects the unwelcoming STEM microclimate to negative mental health due to not fitting in because of how others treat women.

Seven students (24%) described not fitting in their department. Some students felt the STEM microclimate was unaccepting and therefore chose not to connect with people in their department for self-preservation. Three students, an Asian gay man, a white lesbian woman, and an American-Iranian queer transgender individual said that they did not fit in their department nor in SGM spaces. Two students, a Black bisexual woman and the previously mentioned American-Iranian queer transgender individual, Afra, described how their poor feelings of fit led them to consider changing their majors and their schools. Afra explained switching universities:

My trans identity wasn’t respected like my physical disability wasn’t respected. I didn’t feel like there were many resources. It became this compounding set of reasons why I couldn’t take it. I had to leave. It was a really good school too, a top ranked school.

Poor fit caused another student to transfer to a different university as well. Jon, a white gay man, explained that he ended up changing roommates because of homophobic comments by his roommate. Jon noted, “One of the main reasons for me coming to [my university] was the homophobia [at my previous university].”

Collectively, these excerpts demonstrate that reactions to sexual, gender, and racial and ethnic identities within the STEM microclimate affected student perceptions of fit, and that these and other identities, such as disability, intersected to further affect fit. Notably, some students who reported fitting in later went on to also report experiencing microaggressions.

### Experiences and stereotypes greatly affect fit

Given, in part, to reactions to the often heightened visibility of their identities, gender minority (GM) students experienced poorer fit in comparison to sexual minority (SM) students. GM students reported experiencing negative treatment from others when they did not adhere to cis norms regarding clothing and makeup, a lack of appropriate pronoun use by faculty, and discussions of gender-neutral bathrooms by others that were sometimes negative. Jokes, negative comments, and stereotypes about their identities were reported by SM students as well.

#### Clothing

For instance, Carl, a white gay queer non-binary genderqueer student, discussed how they felt rejected by a favorite professor, who had actually gotten them interested in STEM, when they wore makeup and clothing constructed as feminine. The student shared:

I’d gone to lab with my stockings on…. [This professor] said, ‘Oh, Carl, you’re wearing stockings and lipstick…. Is it a costume?’ I’m like, ‘No.’ And he said, ‘Did you lose a bet or something with your friends and you had to wear this? … Well, you know it kind of makes you look like a girl, right?’ I didn’t know what to say, I was getting choked up…. He was laughing about it and noticed I wasn’t laughing and was getting upset so he backed off. He avoided me for the rest of the class. I just went home and cried.

Carl’s experience served to alienate him from his once favorite professor, and, as we show below, impacted his future behaviors regarding presenting as masculine in lab. Similarly, Cathy, a queer Asian woman, told a story about a class experience she had heard about from her mentor regarding clothing. Cathy said:

One of the engineering professors told the class that if you wear a suit, you’ll get extra points. It was kind of isolating to the women who may not want to wear a suit…. It was just this male stereotype comment that excludes women.

In this case, cis-gender expectations about clothing have direct implications for attainment through the connection of grades to cis-gendered clothing expectations by the professor.

#### Pronoun use and other linguistic microaggressions

Just as “feminine” clothing and makeup on students not perceived as women were rejected by others in the STEM microclimate, so too were GM students’ pronouns. For instance, Kim, an Asian bisexual non-binary genderqueer student, described a conversation that they had in an engineering class when their professor explained why she did not agree that a student’s ‘preferred’ pronouns should be used. The student shared, “[The professor said], ‘I don’t agree with saying pronouns…. Trans people are an anomaly and I don’t think we should be normalizing what they do.’” Kim later shared another encounter with a different professor, “It was the first or second day [of class], and the professor said, ‘Non-binary people are an enigma to me.’” Similarly, Alexa, a white bisexual queer woman, talked about how her non-binary friend’s professor rejected their pronouns, “The entire school year [the professor] wouldn’t use the correct pronouns for them, and it made me really angry.” Similarly, when Haruto, an Asian transgender gay man, was asked if he thought any of his identities mattered to any of his instructors or mentors, he discussed an instance where a professor treated him differently. Haruto recalled:

I had a professor that when he found out that I was transgender, he would misgender me persistently. I know he tried not to, but it would slip out and it was a hard situation for me because I have a difficult time correcting him… [because] of the power differential.

Thus, from pronoun use to comments showing prejudice, GM students experienced a linguistic minefield in which their identities were delegitimized in STEM.

#### Bathroom access

In addition to discussions about student identities, participants talked about how others in their STEM department did not accommodate GM identities, such as through the provision of gender-neutral bathrooms. For instance, when asked about their transgender identity and feelings of fit within their department, Michael discussed the role of gender-neutral bathrooms:

They started taking out men’s bathrooms and women’s bathrooms… and made it inclusive. There was some backlash…. People were upset that we were spending money on renovating these bathrooms for trans or non-binary individuals. They felt uncomfortable with male students coming into their bathroom or vice versa.

Afra also talked about the issues they faced regarding gendered bathrooms at their STEM research assistant job. Afra noted:

There weren’t any bathrooms that were genderqueer or single stall. It was uncomfortable because I’m here doing science but I have to have this awkward conversation with my PI…. I told him that the male bathroom is what aligns with my identity…. I had to use the women’s bathroom when I’m on my period and the men’s bathroom when I’m off my period…. [When I used the men’s] I had somebody say something and people stared.

Afra recalled similar issues in their STEM department, though they noted their department was “trans friendly”, with a trans professor and other trans people. Afra shared:

There are opportunities to put in genderqueer bathrooms throughout the… physics building. It’s an old building and has a bunch of single stall bathrooms that are gendered and for the life of me, I can’t remember why they still haven’t switched over…. The building proctor has tried to switch them over, but they haven’t been successful.

#### Jokes, slurs, and stereotypes

Like GM students, SM students reported hearing and experiencing jokes, comments, and stereotypes about their identities. For instance, SM students noted that people often mistook their sexuality or wrongly assumed a hetero identity. For instance, in terms of others not understanding her identity, Tay, a black bisexual woman, explained, “Some of my guy friends, if I tell them I’m bisexual then they say, ‘Well, you’re gay.’ And I’m like, ‘That’s not what that means.’ They’ll say, ‘Yes, it is.’ I’m like, ‘That’s not exactly how it works.’” In another example, Julie, a white lesbian woman, described how her professor made a microaggressive assumption about her sexuality. Julie noted:

I had one professor, we were on a day field camp and I said, ‘A nice cold beer sounds good after this hot day in the sun’. And he said, ‘You’re going to make a man very happy someday’. And I said, ‘Or a woman.’

Homophobic remarks came from both students and faculty. Such remarks included calling undesirable things “gay” as well as comments about students’ clothing. For instance, when asked about insulting comments or jokes, Cathy shared:

I’ve definitely heard people in class talking about things. Saying things like, ‘Oh, that’s really gay’ or just that kind of slam.

She later explained how she heard these kinds of comments in the engineering buildings. Additionally, Jon discussed how his former roommate would often exhibit homophobic language. In one instance his roommate called people homophobic slurs over a videogame microphone. The student shared, “We’ve had little chats about it, how I wasn’t comfortable about that and he continued to do it. So I just said, ‘Okay, I’m going to remove myself from this situation.’”

Students also reported that others held stereotypes about how SM students should behave and speak. For example, Jeff said the opinions of two of his peers changed after they discovered he was not straight. He shared, “It changed their opinion of me because they really thought I was straight… It’s kind of hard to see [because I’m straight passing] … When I told them, I had to explain just because you’re gay it doesn’t change how you act.”

These comments from SGM students show that students experience microaggressions in a variety of STEM settings and from a range of STEM actors. However, some students had alters who helped them cope with these experiences.

### The effect of network-based and participatory social capital on fit

From steering students into career plans to guiding them on classes and internships, students with access to friends, professors, advisors, and organizations that were accepting of SGM identities reported that they fit in STEM better. Students without such social capital described poorer feelings of fit. Frequently, students reported that women and people of color were more welcoming, and felt that they provided better support for fit than older white men, whom students reported often made negative comments and assumptions about SGM students.

#### Differences in faculty support: Women, men, and homophilous alters

Many SGM students reported feeling comfortable talking about their identities with network-based alters who were SGM students and faculty, as well as their closest friends. Eighteen of the 29 students (62%) went to SGM students for emotional and academic support. Friends, both in students’ STEM microclimate and outside of it, provided support that was useful as students worked toward their STEM goals. For example, Kevin described how his friends offered him both academic and social support:

I have a group of friends who I took the scholarship course with. I watched them and learned how they interacted. They’re where I draw a lot of my energy in terms of when I’m needing social support and having academic needs. If I’m having a bad week, I text my friend and we get together and have coffee.

Fourteen out of the 29 students (48%) reported feeling more comfortable talking to and being around women peers and faculty in STEM. For instance, when asked about role models in STEM, Jon, who reported fitting in well and being comfortable in his engineering department, brought up the role of a woman professor in helping him reach his STEM goals. He said:

The professor there, we’ve had a semi-friendly relationship throughout the course of the class. She was the one, if it wasn’t for this class and her as an instructor, I don’t think that I would’ve wanted to go into the oil industry and see if I could make an impact. She definitely helped steer me in that direction after I graduate.

Similarly, Alexa discussed her relationship with a professor around whom she is comfortably open about her sexuality. She commented:

We talked about being a woman in STEM and her experience with that. I feel for me and at my school in general there’s a lot of conversations about women in STEM going on.

Likewise, Cathy talked about leaning on other women of color with the lack of queer people in bioengineering:

I was the only Asian woman there, and there were three other women in the class of thirty people. It was intimidating at first, but as I progressed within my major and took upper level classes and met more people in my major, I’ve become more friends with other women of color in engineering. There’s not a lot of queer people in bioengineering and not many that I take classes with, and so most of my friends are other women of color.

Students who reported that their faculty were accepting described better feelings of fit. On the other hand, students who did not feel accepted described weaker feelings of fit.

In contrast to their relationships with women faculty, students described men faculty as less personal, and noted that they often exhibited behaviors or said things that caused students to pull back or withdraw from the relationship. For instance, Julie talked about experiencing microaggressions from professors who were men, noting:

There’s an unspoken tone of ‘it’s a men’s place, they run the place’, right? I’ve noticed this professor, every time I talk to him he makes this face. I had my closest study partner, he’s male and six-foot-three with blonde hair and blue eyes. If the two of us go to see this professor together he never makes that face. I’ve tested it.

Similarly, Cathy described how she felt neglected in her bioengineering department that is 48% women students but still run by older white men. She noted:

I think a lot of the professors, all the instructors, are men and usually not men of color. They’re very old. It’s harder to talk about your problems or you’re afraid of coming off as like a stereotype. It becomes hard to ask questions and be more involved in the class.

Likewise, Amanda, a white bisexual queer woman, talked about how assumptions around women in engineering pushed her toward women alters. She commented:

It’s made me seek out women more. Most of my friends in engineering are women. … With some of my male professors, I feel more pressure and uncomfortable going to their office hours. I don’t want them to think less of me because of the questions I ask. I don’t really feel that going to my female professors’ office hours, even in STEM.

These comments about men faculty illustrate why SGM students would want to especially seek out women faculty and peers.

#### Professional societies as a source of homophilous alters

Students also talked about how participating in organizations, including those that were SGM-focused inside and outside of the STEM microclimate, helped with emotional and academic support. For instance, students reported going to professional societies for support, a comfortable space where they could be themselves, to network, to look for opportunities, and to talk to other people like themselves. For example, Jess talked about her involvement in non-STEM-focused SGM extra-curricular groups and how they gave her a space to express and discuss her SM identity:

I am a part of a LGBTQIA club at my college. My identity is more present just because you can have that open facilitation of conversation more so in that environment. And then with my closest friends I’m very comfortable talking with that component of my identity.

Similarly, while considering whether she should come out, Cathy described her experience at her school’s LGBT center. She said, “I was interacting with the people in the LGBT equity center and coming to discussion groups. It was very easy to be comfortable and be myself.” In another example, Tay, who reported fitting in well, described the relationships and groups that she relied on to help her achieve her STEM goals:

I rely on my school’s resources. We have a very good career education department and I’ve been talking to them since freshman year about my goals in life. They’re very well informed; obviously a lot of students have gone through there.

As shown, both STEM and non-STEM organizations and spaces play an important role in STEM fit.

### Micro-defenses: Self-presentation

Students employed micro-defenses in two main ways: through self-presentation and by cultivating social networks. First, because how they are treated by others is so crucial to fit and persistence, and because it is not possible for students to avoid all people and interactions in their STEM microclimate that were not SGM-welcoming, students fine-tuned how they self-presented within their STEM microclimate to manage or prevent mistreatment from others. Students used micro-defenses: micro-level interactions through which they managed others’ perceptions of their selves through a range of behaviors, including what they wore, how they spoke, the information they shared about themselves, and even how they used external SGM signifiers (e.g., PRIDE stickers). Students also carefully considered what information to share and used language covering or projecting their sexuality as well as being silent or partially open when it was most useful for them, thus managing who became part of their social network. They also specifically chose to engage professors who were women and people of color to further control the building of a social network containing supportive alters.

#### The use of cis-gender markers in lab and class

GM students discussed STEM microclimate expectations that suggested masculine identities were safer in comparison to feminine ones in many parts of the STEM microclimate. Students who encountered negative attitudes and ignorance about their identities as described earlier often responded with behavioral micro-defenses related to appearance, including wearing gender-conforming clothing and accessories. The goal of these defenses was often to blend in. For instance, Kevin described how he managed his identity in the physics department by presenting as masculine and removing nail polish. He shared:

I put on a masculine face… For St. Patrick’s Day we paint my fingernails this sparkly green color, and the next day I went in to take an exam and forgot to wash it off. I remember feeling, ‘Oh wow, this is not how I normally behave in physics’…. With non-physics friends, I’m open with my sexuality…. I’m less masculine.

Similarly, Carl talked about the calculations they must make about how they present their self when asked about the attitudes of people in their biology department, noting the impact on presentation of self on their grades. Carl shared:

It’s part of life for me to think about feeling safe and comfortable, how I present myself, and how I talk about it to other people. It can affect everything from class, ‘What do I wear to that class? Who do I talk to in that class? How talkative am I?’ It affects my grades. I worry in classes where professors single [me] out for my identity…. I’m conscious about the way I look… when I know I have lab and choosing what to wear.

Likewise, when asked if they ever felt excluded from any communities on campus, Carl continued that they used a strategy of presenting masculinely in lab to avoid ‘bad interactions’. Likely resulting from their traumatic experience with a once-favorite professor, they said:

I’ve been trying to get better about this, but I only present as male in lab. I don’t know what it is about the lab environment. Something about it makes me uncomfortable and I’ve had enough bad interactions with people in labs to persuade me that presenting male and honestly acting pretty straight is the way that I have to go about existing in a lab.

These quotes illustrate the lab space as a climate zone in the STEM microclimate that is less accepting of SGM identities and the work students do to avoid mistreatment from others. In addition to managing how others perceived their appearance, behavior, and demeanor, students used micro-defenses in the ways they externally crafted their SM identities. For instance, Amanda recounted that she made calculations about ways to share her identity through the use of signifiers outside the body, such as stickers on her laptop. Her concern that unaccepting professors might react to pride stickers on her laptop negatively played a role in her decision about how to decorate it. She said:

I have some different stickers that have a bi-pride flag on them, but I don’t really feel comfortable putting them on the outside of my laptop. I think [if I open up my laptop] in class… it’s hard to know how a professor would feel about it. So I just don’t quite feel comfortable doing that.

The cases show how behavioral micro-defenses were used to protect identity information in instances when external signifiers, including clothing/makeup, demeanor, or even stickers, could reveal information about SGM identities.

#### Sharing information with the trustworthy

Outside of material signifiers, students also utilized oral micro-defenses that sent messages to others about their identity. For instance, when asked about managing identities when interacting with people in her STEM program, Amanda talked about referring to her significant other as her “friend” rather than her “girlfriend.” Other times, having other people know about her girlfriend/sexuality provided an armor against unwanted sexual advances by men in her STEM microclimate. She said:

Sometimes I want to tell a story about my girlfriend, but I don’t always feel comfortable saying that, so I’ll say ‘friend.’ But in some cases, it’s kind of helpful because if I’m studying with guys late at night and I don’t want them to start hitting on me, then I’ll bring up my girlfriend and then it’s really easy.

Alternately, Lou, a black bisexual woman, explained how she remained silent when topics of identity were being discussed to try to avoid microaggressions. She commented:

When people have conversations about things, I don’t really… share information about myself in those situations…. Most of the time it’s just small comments or it’s microaggressions…. Sometimes professors, sometimes from people in the class, things like that will happen. They’re very small but then add up towards the end.

Thus, sharing identity information orally was a micro-defense that students used in order to exert control over how others perceived them and potentially avoid negative reactions.

Students often reported wanting to share private information only with those they could trust or around whom they were comfortable. For instance, Chris, a white gay man, described sharing personal information with those he is close to:

When it comes to speaking with peers, I would have to know a little bit about them or have spent five or ten minutes in the same room as them before I would want to be open about it…. There will be people you run into where it’s best if I just don’t bring that up.

Ash, a white lesbian woman, similarly described managing her identity around instructors. Ash said:

I don’t talk about [my sexual identity] unless it’s relevant…. I feel confident talking about it when I know the instructor has experience with the LGBT community…. So I don’t talk about it unless it’s an instructor I know will be okay with it and it won’t affect my academic life…. I don’t discuss my personal life and it is in fear that it will come back to haunt me.

Together, these comments demonstrate the broad range of practices students engage in to craft an identity that their experience has suggested will be safer in STEM.

### Micro-defenses: Cultivating a safe social network and microclimate

As mentioned, the second main micro-defense that students employed entailed a deliberate cultivation of social networks and STEM microclimates in attempts to surround themselves with friendly people and avoid negative interactions, and indeed to increase their fit and promote persistence. These students engaged in a variety of sophisticated and extensive measures designed to yield a STEM microclimate terrain that included accepting people and avoided those who were unaccepting. Students engaged in cultivation strategies when they chose what classes or universities in which to enroll, ‘testing the waters’ to determine if people were accepting, and seeking out women and people of color as mentors.

#### Choosing safe universities and classes

In some cases, students picked what classes they would take based on how many SGM students would be there and whether the professors were accepting. For instance, Carl talked about avoiding unaccepting professors. They said, “Sometimes you really want to take the class and so if [the professor is] kind of shitty you’re willing to take that risk. But there are definitely people from the stories I’ve heard that I would never take their class.” Unfortunately, by avoiding professors and other situations, students can miss out on opportunities and suffer academically. Carl continued to suggest that avoiding unaccepting professors negatively impacted their grades. They shared:

[I have some] bad grades because I was discouraged, I wouldn’t go to a professor’s office hours if I don’t feel like they would support me…. Most people would not sympathize with me if I said, ‘Oh I had a transphobic professor’…. [My concern is to] first and foremost to feel safe and secure.

In addition to choosing classes by how accepting the professors teaching those classes are, some students chose their university based on how accepting it is towards SGM students. For instance, Kim said:

I knew these other people who were trans and queer… and some went to state schools or went to school in the south. They had a lot more difficulty with their identity than I ever had. I chose to go to [my university] because I knew it was known as the gay Ivy, that people there were generally pretty liberal. I did that to protect myself to begin with.

Kylie shared a similar experience. She said, “I was pretty careful about picking out a school where I could be openly gay, that was really important to me picking out a school.”

#### Testing the waters

In order to determine when it was safe to share information about identities or when micro-defenses might need to be deployed, students reported “feeling out” or “testing the waters” in interactions with new people to determine how safe or accepting they might be of SGM identities. They could then avoid unaccepting people or avoid potentially problematic situations. For example, Kylie said:

I bring up [my identities] with people the first time I meet them. … If someone isn’t going to be accepting or friendly, I want it to be within my first two weeks of class so I could drop the class. I’d want it to be within the first couple times hanging out so I don’t build a friendship with someone who is homophobic. I bring it up early is my strategy.

Similarly, Kim shared, “[My mentor told me that she heard that] there’s a grad student in my department who says, ‘I don’t think gay people should be allowed to marry’…. Something to that level hasn’t happened to me, I go out of my way to avoid interactions that can cause that.”

#### Seeking alters who are women and people of color

Given that students reported that they felt more comfortable with women professors and peers, students actively sought to surround themselves with such alters in their STEM microclimate. In a field that is limited in diversity, minority STEM SGM students tended to seek out others who were similar to them who could relate to their experiences and offer support. For instance, Kim had this to say when asked about their social network:

I prefer to do homework with girls or with mixed groups because I feel like personality wise, a lot of girls are less likely to try to act overconfident and speak over you.

Similarly, Mackenzie, a white bisexual woman, searched for a woman professor to be her mentor. She said:

She leads a lot of programs, she does conferences, things like that. So I did apply to work with her and actively sought out her mentorship…. Just being a woman in science, being a queer woman in science, it’s nice to actually have those associations.

Additionally, Kim discussed ‘consciously’ seeking out a minority advisor to defend against uncomfortable environments. The student said:

I was emailing professors and asking if I could work in their labs. I definitely tried to talk to professors of color or women professors first because I knew I would feel more comfortable in their environment. So that’s been a conscious choice I made.

Likewise, Cathy said, “I don’t know if I do anything consciously, maybe subconsciously I interact with more women and kind of just stay within my bubble because I’m weary of interacting.” Further, Lou talked about her considerations when choosing her advisor. She shared:

One of the most important choices was choosing an advisor and figuring out who in the department was good to talk to about which classes to take or what internships to do…. [My advisor is] the only female teacher in the department and she’s someone I just met. I took her class last semester, but she’s been awesome and responsive when I’ve asked about anything…. All the time I think about finding someone in the department who might understand my past experiences or how I might be feeling at the particular moment being a student at the college…. There’s not much diversity in the department [which] affected my choices [of an advisor and] limited what I have to choose from.

These comments show the complex calculations and behaviors in which SGM students engage to create a safe STEM microclimate for themselves in which they cultivated SGM-welcoming social networks that helped them circumvent people, programs, and classes that were incongruent with or hostile to their identities.

## Discussion

By addressing the four research questions in this study, we offer an initial unpacking of the complex relationship between the STEM microclimate, fit, social capital, and micro-defenses—all of which can affect STEM persistence. Students’ perceptions of how they fit, the comments they heard that affected their fit, the people to whom they reached out, and the micro-defensive mechanisms in which they engaged are impacted by perceptions of how intersectional identities fit within the constellation of sexual, gender, and racial/ethnic identity hierarchies in STEM. While many (41% of participants) reported fitting in, a quarter of students fit in only sometimes. Another quarter of students did not fit in, while about 14% did not explicitly address fit in their comments. Further, even when students noted that they fit, many still reported experiencing microaggressions.

Generally, hearing about or experiencing stereotypes relating to gender or sexuality was reported as affecting participants’ feelings of fit within their STEM microclimates. The addition of microaggressive acts against STEM students with SGM identities, including the assumption of heterosexuality [43] and cisnormative identities, further complicated students’ ability to fit into their STEM departments. As participants discussed, these acts came from both STEM peers and professors. Our work finds that faculty use of correct student pronouns continued to be an issue for GM students in the STEM microclimate, supporting earlier work suggesting it was a problem [14]. Additionally, participants’ reports of negative treatment by others for self-expression that did not adhere to traditional gender norms (e.g., people perceived as men wearing nail polish), particularly an issue in STEM labs, offers additional nuance to McGee’s identification of climate zones in the STEM microclimate [29] and corroborates and extends Hughes’ [50] work on the effects of hegemonic masculinity with engineering students. Importantly, microaggressions to the often hypervisibility of GM students’ identities appeared to be connected to lower feelings of fit, and SGM issues of fit were especially compounded for racial/ethnic minority students.

Social capital in the form of social networks appeared tied to students’ reports of good or poor fit. This supports McGee’s assertion that a lack of SGM alters can affect student fit in the STEM microclimate [29]. Connected to the previous point that intersecting identities affected how students are treated by others, students felt more comfortable around individuals with minoritized statuses, in this case women and racial minority faculty and peers, and actively sought them out. Supportive professors offered safe spaces, emotional support, and advice that helped students in the STEM programs, as found in previous research [66]. Unfortunately, given the low levels of diversity in STEM, students sometimes must persist through years of initial classes to finally find an alter who is not homo/transphobic, at least overtly. This is a middle road strategy that many reported choosing.

SGM students actively implemented defenses against discrimination, including those that we call micro-defenses—small-scale interactional behavior they displayed to better position them to defend themselves against mistreatment from others. By introducing the concept of micro-defenses, we broaden theory about interactional processes managing fit and STEM persistence, including those drawing upon pre-existing work on microaggressions [e.g., 11, 33, 70], reactions to microaggressions [e.g., 13], social capital theory, and the agency through which students cultivate their networks. There were clear connections between the reported microaggressions (e.g., mistreatment by faculty for students wearing clothes faculty thought were not gender-conforming) and the nuances of the defenses (e.g., presenting as masculine in lab). Students provided examples of specific, micro-level behavior in which they engaged to speak back (figuratively or literally) to microaggressions they experienced, or used in an effort to prevent experiences related to “chilly” microclimates [19, 20, 43, 75]. These defenses included engaging in micro-interactional self-presentation of behaviors in specific places and avoiding problematic people or cultivating their social networks to seek out allies.

Crucially, we found that participants often expanded their networks, sought out people, and engaged in relationships with women, people of color, or with people with whom they were homophilous—individuals like themselves. SGM students made important academic decisions based on their perceptions of acceptance and support by those in their social networks. This support helped students avoid unaccepting people when possible. This finding expands previous work showing that women held more welcoming stances toward minoritized groups [66], and that trans students engage in efforts to determine how accepting a university is before choosing to attend [76]. We show that SGM students were aware of how they are viewed by different groups vis-à-vis various intersecting hierarchies, and deployed this information to surround themselves with such individuals to promote their own success. This likewise complements McGee’s work showing that students actively work to create a network that will be supportive for their identities [29]. This finding also broadens previous work by showing that students’ toolkit of defenses goes beyond trying to fit into the heteronormative climate by changing how others perceive them (e.g., passing) to changing their social network in their STEM microclimate [68]. This means that these SGM students who persist have likely positioned themselves to succeed by building a support system that will promote their persistence.

## Conclusion

This research has highlighted how SGM students engage in micro-defenses relating to presentation of self and crafting social networks to support their success—surrounding themselves with safe, supporting people because their STEM microclimate as a whole was not supportive of their persistence in STEM due to others’ reactions to their SGM identities. It featured gender minority students’ experiences, particularly important given that literature on trans and non-binary students in STEM is nascent; thus strengthening the theoretical contribution of the work. STEM persistence for minoritized groups is unlikely to increase without structural change within the programs and institutions, rather than focusing on change among minoritized students, who are already doing the heavy lifting [29].

Because the STEM microclimate is generally cis- and heteronormative, and feelings of fit support persistence, it is important that SGM students have the resources to help them feel that they fit in their departments. Such resources include network-based and participatory social capital—SGM faculty, organizations, and peers—that can provide guidance, support, and insight to these students to enhance their ability to persist in their field [32, 34, 35, 55]. Less than half of the SGM students interviewed felt they completely fit in their department, while over half of the sample were affected by negative stereotypes and behaviors that contributed to poorer feelings of fit. The gap between how students who fit and did not fit felt about their departments, faculty, staff, and students within the STEM microclimate needs to be minimized to increase the persistence of a diverse group of students and ideas in STEM. Barriers to these efforts exist in departments wherein SGM identities are not yet commonly considered part of diversity initiatives [50].

Too often, suggestions for improvement point to ways that minoritized individuals can better survive in their toxic programs, without also addressing the needs and ways that programs and people with majority identities can change to facilitate warmer climates [29]. Given that STEM microclimates are often chilly for SGM students, and that previous research has documented that SGM students consider “faculty in STEM… ill-equipped to deal with their unique issues or unable to connect with them on more than a superficial level” [26, p. 15], the findings presented in this study highlight the importance of structural interventions by STEM program administrators. Such interventions should focus on programmatic changes making STEM programs more welcoming to SGM students through education opportunities for existing STEM faculty and students, particularly those who do not have SGM identities, that directly address bias and structural injustice [27]. The findings in this research provide numerous examples of ways programmatic changes (e.g., gender neutral bathrooms) and training (e.g., correct pronoun use) can be made. Structural changes must also include the hiring of more SGM, women, and people of color faculty in STEM, and address various climate zones, such as lab and those noted in academic conferences [77]. Forbes offers practical programmatic changes to address the unwelcoming STEM microclimate, including mandatory safe zone training and queer competency education training for faculty, as well as queer affinity groups (e.g., public clubs) in STEM. However, such changes should not disproportionately draw upon the efforts of women and racial minority faculty, and majority faculty can and must offer support to students and be supported in how to do that [26]. A first step is for faculty who are not already aware of the STEM climate faced by SGM students to become aware of it so that they can help address it. Applications in McGee for racial and ethnic minorities in STEM could also be adjusted to support SGM students [29].

Future research should include longitudinal studies to further examine the ways that the four variables of interest in this study connect. These studies should ideally utilize a mixed method longitudinal approach to identify how the variables of microaggressions, fit, micro-defenses, and social networks interact with each other and affect persistence in STEM for students of various SGM and other minoritized identities. Such research with larger sample sizes could investigate intersectional issues faced by SGM students of color and those with additional identities and statuses, including disabilities. Future research should also examine how changes in STEM microclimates can be made to make them more welcoming and encourage student perceptions of fit and their persistence. Such interventions aimed at majority individuals and improving structures should also be undertaken. However, such research and interventions should not be too narrow as to discount university and other contexts outside of STEM that affect persistence for minoritized students. Additional improvements in research on SGM STEM students may help address the problems identified in this study [5]. This includes the expansion of large NSF surveys that have been in place for decades to add in SGM identities, so that data sets addressing SGM in STEM are more widely available. Generally, such expansions must extend beyond these NSF STEM censuses, to considerations of SGM definitions as well as within university and funding organizations’ purview, programs, and data collection [5].

## Supporting information

S1 Protocol Demographic questionnaire. (DOCX)

S2 Protocol Interview guide. (DOCX)
