# Supplementary material for: Sexual and gender minority undergraduates’ relationships and strategies for managing fit in STEM
Source: PLoS One. 2022 Mar 17;17(3):e0263561. doi: 10.1371/journal.pone.0263561 (PMC8929613; doi:10.1371/journal.pone.0263561)
Supplement: S1 Protocol — (DOCX) [file pone.0263561.s001.docx]

Measuring the Effects of Academic Climate and Social Networks on

Persistence of STEM Undergraduates

**Information Form**

Q1. How would you describe your sexuality? (*Select all that apply*)

Lesbian

Gay

Bisexual

Queer

Questioning

Asexual

Heterosexual

If none of the above apply, how would you describe your sexuality? _________________________________________________________

Q2. How would you describe your gender identity? (*Select all that apply*)

Woman

Man

Transgender

Gender NonBinary/Genderqueer

Intersex

If none of the above apply, how would you describe your gender identity? ____________________________________________________________

Q3. What university do you attend?

____________________________________________________________

Q4. Based on your accumulated credit hours, you are a

First year student

Sophomore

Junior

Senior

Other, please explain __________________________________________________

Q5. Your anticipated graduation date is _______________________________________

Q6. You are an International Student (F-I visa) *You are still eligible to participate in this study*

Yes

No

Q7. I am pursuing my STEM goals in: (*Check all that apply*)

Agricultural Sciences (e.g., Animal Sciences, Natural Resources), please specify major _________________________________________________________________________

Chemistry (e.g. Chemistry, Forensic Chemistry), please specify major _________________________________________________________________________

Computer Science (e.g., Computer Science, Management Sciences), please specify major _________________________________________________________________________

Engineering (e.g., Biomedical Engineering, Electrical Engineering), please specify major ___________________________________________________________________________

Environmental Science (e.g., Environmental Science and Environmental Studies), please specify major ________________________________________________________

Geosciences (e.g., Geology, Earth Sciences), please specify major ___________________________________________________________________________

Life/Biological Sciences (e.g., Biology, Neuroscience), please specify major ___________________________________________________________________________

Mathematics (e.g., Applied Mathematics, Actuarial Science), please specify major ___________________________________________________________________________

Physics/Astronomy (e.g., Physics, Astronomy and Astrophysics), please specify major ___________________________________________________________________________

Other, please specify ____________________________________________________

Q8. Other, non-STEM, majors, minors, or concentrations you are pursuing:

_________________________________________________________________________

Q9. What is the highest level of education of your parents?

  ________________________________________________________________________

Q10. You are (*You can select more than one*)

American Indian/Alaska Native -- *Share name of enrolled or principal tribe(s) for example, Mayan, Navajo, Tlingit, and so on.* ____________________________________

Asian -- *Share origin(s), for example, Indian, Chinese, Pakistani, Trinidadian, and so on.* __________________________________

Black/African American --   *Share origin(s), for example, Haitian, Nigerian, Jamaican, and so on. _______________________________________________________________*

Hispanic

Cuban

Mexican, Mexican American

Puerto Rican

Other Hispanic or Latino origin --   *Share origin(s), for example, Argentinean, Colombian, and so on.* __________________________________

Native Hawaiian/Other Pacific Islander --   *Share origin(s), for example, Guamanian or Chamorro, Fijian, Samoan, Tongan, and so on.* __________________________________

Middle Eastern/North African/Arab --   *Share origin(s), for example, Egyptian, Lebanese, Moroccan, and so on.* _____________________________________________

White --  *Share origin(s), for example, German, Irish, French, British, and so on.* _________________________________________________________________________

Other ethnicity, race or origin -- *Share origin(s).* ______________________________

Q11. Your current university email address *(you must provide to receive the electronic Amazon gift card)*: _____________________________________________________________

Q12. Best email address to reach you: ___________________________________________

Q13. Best telephone number to reach you: _______________________________________
