# Supplementary material for: Sexual and gender minority undergraduates’ relationships and strategies for managing fit in STEM
Source: PLoS One. 2022 Mar 17;17(3):e0263561. doi: 10.1371/journal.pone.0263561 (PMC8929613; doi:10.1371/journal.pone.0263561)
Supplement: S2 Protocol — (DOCX) [file pone.0263561.s002.docx]

# Measuring the Effects of Academic Climate and Social Networks

# on Persistence of STEM Undergraduates

# **Interview Protocol**

# 0. Introduction

0.1 What is your STEM major? How did you get interested in that major? Have you ever switched majors or considered switching to a different major? (STEM or non-STEM?) What were some of the reasons?

0.2 When do you plan on graduating? What are you planning to do after graduation? What goals do you have associated with your future STEM degree? (Clarify specifics, including career goals.)

# 1. Demographics and Identities

1.1 Go over demographic form. [Probe for all identities. This should be a conversation to inform on how the survey items should look.]

a. Did this form do a good job capturing how you identify?

b. How could it be improved?

1.2 How “out” are you regarding your identities in your STEM program? [Probe: Peers, instructors, mentors] How do you manage these identities when interacting with:

a. others [Probe: Peers, instructors, mentors] in your STEM program? (if they switched to another major also ask about that department)?
b, communities outside your STEM program? [Probe: family, other personal relationships, extracurricular communities]?

**For ALL individuals ask about pronouns/names they wish to be identified by**

1.3. Do you think any of your identities have mattered to your peers/instructors/mentors in your STEM program? In what ways? [Probe gender/sexuality and positive or negative reactions to “coming out”] [If they switched to another major also ask about that department]?

# 2. Navigating the STEM ideological terrain—Choices & Challenges

2.1. Could you tell us about the most important choices you have made to pursue your STEM goals (choosing mentors, organizations, working with peers)?

2.2. How have others’ perceptions of or reactions to your identities and experiences affected your choices while pursuing your STEM goals? [Probe gender/sexuality] Do you have role model(s) that have affected your choices? How?

2.3. How have you dealt with challenges you’ve faced while pursuing your STEM goals?

# 3. Social Networks

3.1a. What relationships or groups have you relied on, and to what degree, to help you achieve your STEM goals? [If not mentioned, probe if the influencer has a STEM background or what their level of education is.]

3.1b. [For each relationships/groups mentioned] What have these relationships offered you?

a. Tell me about the kind of information and resources you have accessed through these relationships to assist you in pursuing your STEM goals.

b. What kinds of encouragement and empowerment have you received through these relationships to achieve your STEM goals?

3.1c. What other relationships have you relied on? [Ask 3.1b for each relationship] Probe for:

a. faculty mentoring & communication (office hours, networking, research opportunities, etc.)

b. if relevant: relationships in 2^nd^ major

c. peer relationships (study groups, etc.)

d. professional societies/organizations

e. LGBTQIA+ groups/organizations

f. family/friends

g. roommates/suitemates

h. online social networks such as Facebook, Twitter, Reddit

3.1d. In what ways have others’ perceptions of your identities affected these relationships or what you have received out of these relationships? [Probe for intersectionality during this discussion]

3.1e. Have you ever felt excluded within any of these communities?

3.2. What types of support [financial, emotional etc.] and from whom [family, peer, departmental etc.] have you wanted, but not fully received? [academic, emotional]

# 4. Academic Climate

4.1 Are there are others in your STEM program (faculty, staff, peers) who are “out” as LGBTQIA+?

If Yes: a. What does that mean to you?

b. Have you gone to those individuals for support?

c. Is there a community among those who hold LGBTQIA+ identities?

If No: a. Why do you think there are no faculty, staff, peers who are “out”?

b. How might having out faculty, staff, and peers help you?

4.2a As an individual who is [insert ALL their intersectional identities], how do you feel like you fit in your STEM program?

4.2b. To what extent does the climate/environment of your STEM program make you consider changing your major to another STEM major or a non-STEM major?

**Ask questions on their specific sexuality/gender identity first.**

4.3a. Have you **experienced** interactions, comments or jokes that are insulting, degrading, silencing, threatening, or aggressive about those who are **not heterosexual**?

If Yes: a. Could you give us an example or describe what occurred?

b. How did you respond?

4.3b. Have you **witnessed** interactions, comments or jokes that are insulting, degrading, silencing, threatening, or aggressive about those who are **not heterosexual**?

If Yes: a. Could you give me an example or describe what occurred?

b. How did you respond?

4.3c. Have you **heard of** interactions, comments or jokes that are insulting, degrading, silencing, threatening, or aggressive about those who are **not heterosexual**?

If Yes: a. Could you give me an example or describe what occurred?

b. How did you respond?

4.4a. Have you **experienced**, interactions, comments or jokes that are insulting, degrading, silencing, threatening, or aggressive about those who identify as **non-binary or transgender**?

If Yes: a. Could you give me an example or describe what occurred?

b. How did you respond?

4.4b. Have you **witnessed** interactions, comments or jokes that are insulting, degrading, silencing, threatening, or aggressive about those who identify as **non-binary or transgender**?

If Yes: a. Could you give me an example or describe what occurred?

b. How did you respond?

4.4c. Have you **heard of** interactions, comments or jokes that are insulting, degrading, silencing, threatening, or aggressive about those who identify as **non-binary or transgender**?

If Yes: a. Could you give me an example or describe what occurred?

b. How did you respond?

4.5a. Have you **experienced** interactions, comments or jokes that are insulting, degrading, silencing, threatening, or aggressive about those who are **racial or ethnic minorities**?

If Yes: a. Could you give me an example or describe what occurred? Other examples?

b. How did you respond?

4.5b. Have you **witnessed** interactions, comments or jokes that are insulting, degrading, silencing, threatening, or aggressive about those who are **racial or ethnic minorities**?

If Yes: a. Could you give me an example or describe what occurred?

b. How did you respond?

4.5c. Have you **heard of** interactions, comments or that are insulting, degrading, silencing, threatening, or aggressive about those who are **racial or ethnic minorities**?

If Yes: a. Could you give me an example or describe what occurred?

b. How did you respond?

4.6a. Have you **experienced** interactions, comments or jokes that are insulting, degrading, silencing, threatening, or aggressive about **women**?

If Yes: a. Could you give me an example or describe what occurred? Other examples?

b. How did you respond?

4.6b. Have you **witnessed** interactions, comments or jokes that are insulting, degrading, silencing, threatening, or aggressive about **women**?

If Yes: a. Could you give me an example or describe what occurred?

b. How did you respond?

4.6c. Have you **heard of** interactions, comments or jokes that are insulting, degrading, silencing, threatening, or aggressive about **women**?

If Yes: a. Could you give me an example or describe what occurred?

b. How did you respond?

4.7. To what extent do these attitudes and behaviors [in 4.3-4.6] affect your level of comfort or safety in your STEM program, as someone who is [Insert their intersectional identities]?

4.8. To what extent do these attitudes and behaviors [in 4.3-4.6] make you consider changing your relationships in your **STEM program** (e.g., mentors, peers, faculty, study groups)?

4.9. To what extent do (or did) these attitudes and behaviors make you consider changing your **major**?

1. What major were you thinking of changing to? Why?
2. Did you consider transferring to another university? Or leaving university completely?
3. Did you talk to anyone about this? Who? How did they respond?

# 5. Wrap Up

5.1. Overall, how has your STEM program demonstrated inclusiveness or exclusiveness toward LGBTQIA**^+^** students? What could they do to enhance inclusiveness toward LGBTQIA+?

5.2. Is there anything else (that we did not cover) that you want to share about your experiences in STEM?

**Thank you for your participation!**
